# Neurotoxicity of a Biopesticide Analog on Zebrafish Larvae at Nanomolar Concentrations

**DOI:** 10.3390/ijms17122137

**Published:** 2016-12-19

**Authors:** Ahmed Nasri, Audrey J. Valverde, Daniel B. Roche, Catherine Desrumaux, Philippe Clair, Hamouda Beyrem, Laurent Chaloin, Alain Ghysen, Véronique Perrier

**Affiliations:** 1 U1198 MMDN (Molecular Mechanisms of Neurodegenerative Diseases), Inserm (National Institute for Health and Medical Research), 34095 Montpellier, France; a7mednas@gmail.com (A.N.); audrey-valverde@hotmail.fr (A.J.V.); desrumauxcatherine@yahoo.com (C.D.); veronique.perrier@umontpellier.fr (V.P.); 2 BioCampus, University of Montpellier, 34095 Montpellier, France; 3 EPHE (Ecole Pratique des Hautes Etudes), 75007 Paris, France; 4 Laboratory of Environment Biomonitoring, Faculty of Sciences of Bizerta, University of Carthage, 7021 Zarzouna, Tunisia; 5 IBC (Computational Biology Institute), CNRS (National Center for Scientific Research), University of Montpellier, 860 rue Saint Priest, 34095 Montpellier, France; daniel.roche@lirmm.fr; 6 CRBM (Research Center for Cell Biology in Montpellier), UMR 5237, CNRS, 1919 route de Mende, 34293 Montpellier, France; hamouda.beyrem@gmail.com; 7 MGX (Montpellier GenomiX), BioCampus, University of Montpellier, 34095 Montpellier, France; philippe.clair@univ-montp2.fr; 8 CPBS (Center for Study of Pathogens and Biotechnologies for Health), FRE 3689, CNRS, University of Montpellier, 1919 route de Mende, 34293 Montpellier, France; laurent.chaloin@cpbs.cnrs.fr

**Keywords:** α-terthienyl, pyrimidine, mechanosensory organ, posterior lateral line, mechanosensory system, hair cells

## Abstract

Despite the ever-increasing role of pesticides in modern agriculture, their deleterious effects are still underexplored. Here we examine the effect of A6, a pesticide derived from the naturally-occurring α-terthienyl, and structurally related to the endocrine disrupting pesticides anilinopyrimidines, on living zebrafish larvae. We show that both A6 and an anilinopyrimidine, cyprodinyl, decrease larval survival and affect central neurons at micromolar concentrations. Focusing on a superficial and easily observable sensory system, the lateral line system, we found that defects in axonal and sensory cell regeneration can be observed at much lower doses, in the nanomolar range. We also show that A6 accumulates preferentially in lateral line neurons and hair cells. We examined whether A6 affects the expression of putative target genes, and found that genes involved in apoptosis/cell proliferation are down-regulated, as well as genes reflecting estrogen receptor activation, consistent with previous reports that anilinopyrimidines act as endocrine disruptors. On the other hand, canonical targets of endocrine signaling are not affected, suggesting that the neurotoxic effect of A6 may be due to the binding of this compound to a recently identified, neuron-specific estrogen receptor.

## 1. Introduction

The 20th century was marked by the tremendous development of heavy petrochemical industry that led to the annual production of 500 million tons of chemical derivatives distributed as follows: 300 million tons of synthetic compounds used in industrial and consumer products, 140 million tons of fertilizers and about 5 million tons of pesticides [1,2]. Contamination of freshwater systems with thousands of industrial chemical compounds, especially fertilizers and pesticides (herbicides, fungicides, insecticides) entering into the food chain, is one of the key environmental problems facing humanity. Although most of these compounds are present at low concentrations (μg/L to pg/L), many of them raise considerable toxicological concerns, with largely unknown long-term effects on aquatic life and on human health. For instance, many studies showed that contaminations of rivers with estrogenic and androgenic compounds in the ng/L range, are responsible for intersex changes within fish populations [3,4] raising concerns about population-level consequences [5]. In Brazil, a study conducted in 2710 newborn males revealed fetal contamination with organochlorine pesticides (DDT) with a risk factor for external genital malformations [6], whereas in young girls, fetal and/or environmental exposure to endocrine disrupting chemicals (EDCs) led to precocious puberty [7,8].

On the other hand, many species of arthropods have become increasingly resistant to conventional insecticides, a situation that is particularly worrying when considering the mosquito vectors of tropical diseases [9,10]. The search for new generations of insecticides is therefore a necessity [11]. Over the last decade, researchers and industrials have developed “green” chemistry which aims at designing more environmentally friendly industrial processes, and at generating more benign products [12]. Besides, the arrival of new technologies such as high throughput screening and mass spectrometry in the 1990s boosted “green” chemistry, i.e., the identification of new molecules extracted from plants, and the production of derived molecules with improved properties [11,12,13].

One such molecule is A6, a putative insecticide that was derived from the naturally occuring pesticide α-terthienyl, a molecule isolated from the roots of the common marigold (*Tagetes erecta*) [14,15]. In A6, one of the thienyl rings of α-terthienyl has been substituted by a pyrimidine, making it structurally related to another class of pesticides, the anilinopyrimidines, which are widely used to protect plants from the cryptogamic fungus, *Bothritis cinerea* [16]. In a previous study, we showed that A6 is able to induce the formation of sodium dodecyl sulfate (SDS)-resistant oligomers of the misfolded prion protein, called PrP^Sc^, in a prion-infected cell line [17,18,19].

In order to better understand the molecular effects of A6 and anilinopyrimidines on living animals, we chose to work on zebrafish larvae because of their many experimental advantages, including transparency of the body, and availability of transgenic lines that allow one to visualize various cell types in vivo. We found that A6 is highly toxic for zebrafish larvae and induces degeneration of their central nervous system. The anilinopyrimidine cyprodinyl also affects survival, though less markedly than A6, and to a minor extent, neuronal integrity.

We then focused on the lateral line system, a sensory system specific to fish and amphibians but closely related to the mammalian inner ear, to better quantify the neurotoxic effects of A6. We observed that very low doses of A6 have a clear effect on axonal and hair cell regeneration. Gene expression analyses and molecular modeling studies of A6 and cyprodinyl support the idea that both compounds may bind to androgen/estrogen receptors, consistent with previous reports that anilinopyrimidines act as endocrine disruptors [20]. Our results raise the question of the relationship between endocrine disruption and neurotoxicity, and lead us to suggest that binding of pesticides to estrogen receptors may have direct neurotoxic effects.

## 2. Results

### 2.1. A6 and Cyprodinyl Induce Lethality and Behavioral Defects in Zebrafish Larvae

**Figure 1:**
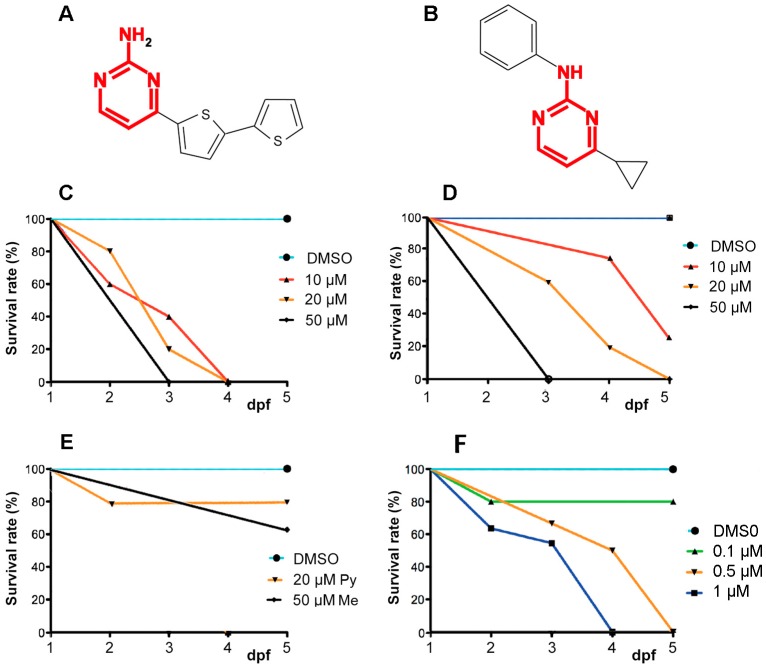
Effects of pyrimidine-containing pesticides on survival of zebrafish larvae. (**A**,**B**) Formulas of the compounds A6 and cyprodinyl, respectively. The pyrimidine moiety has been highlighted in red; (**C**,**D**) Survival curves at 2–5 days post-fertilization (dpf) of wild-type embryos exposed at 1 dpf to various concentration of A6 (**C**) and cyprodinyl (**D**); (**E**) Survival curves for pyrimethanyl and mepanipyrim, survival was 100% at all concentrations except 20 μM pyrimethanyl and 50 μM mepanipyrim; (**F**) Survival curves for very low concentrations of A6. Each point represents the pooled data from two independent experiments (*n* = 10–20 larvae). Differences between the survival curves in panels (**D**,**F**) are statistically significant (non parametric Mantel-Cox log-rank test, *p* = 0.00119 for (**D**) and *p* = 0.048 for (**F**)).

Zebrafish embryos were exposed to A6 (Figure 1A) or to three anilinopyrimidines, cyprodinyl (Figure 1B), pyrimethanyl, and mepanipyrim at concentrations ranging from 10 to 50 µM, from 1 day post-fertilization (dpf) onwards, and their survival rate was determined at 2–5 dpf. In the control group, 0.1% dimethyl sulfoxide (DMSO) did not induce any mortality or deformity, as already described [21]. A6 turns out to be highly toxic, as all larvae died after 3 days of incubation at 10 µM (Figure 1C). Cyprodinyl is also toxic, though to a lesser extent than A6, with less than 20% survival after 3 days, and 0% after 4 days of incubation in a 20 µM solution (Figure 1D). The two other anilinopyrimidines tested have a much lower effect, if any, on larval survival (Figure 1E).

We observed that larvae treated with either A6 or cyprodinyl display abnormal behaviors (uncoordinated swimming, lack of response to water flow, immobility etc.). When put in an observation chamber for 10 min, normal embryos display active swimming mostly confined to the edges of the dish (Figure S1). Embryos exposed to 20 µM cyprodinyl for 3 days are mostly immobile (Figure S1, duplicates in upper row). As all embryos exposed to 20 µM A6 were dead after 3 days of treatment, we exposed them to a lower concentration (50 nM, duplicates in the lower row of Figure S1) and observed again abnormal behaviors, with the embryos remaining confined to part of the dish, and spending more time in the open space at the center.

### 2.2. Effect of A6 and Cyprodinyl on Spinal Cord Neurons

**Figure 2:**
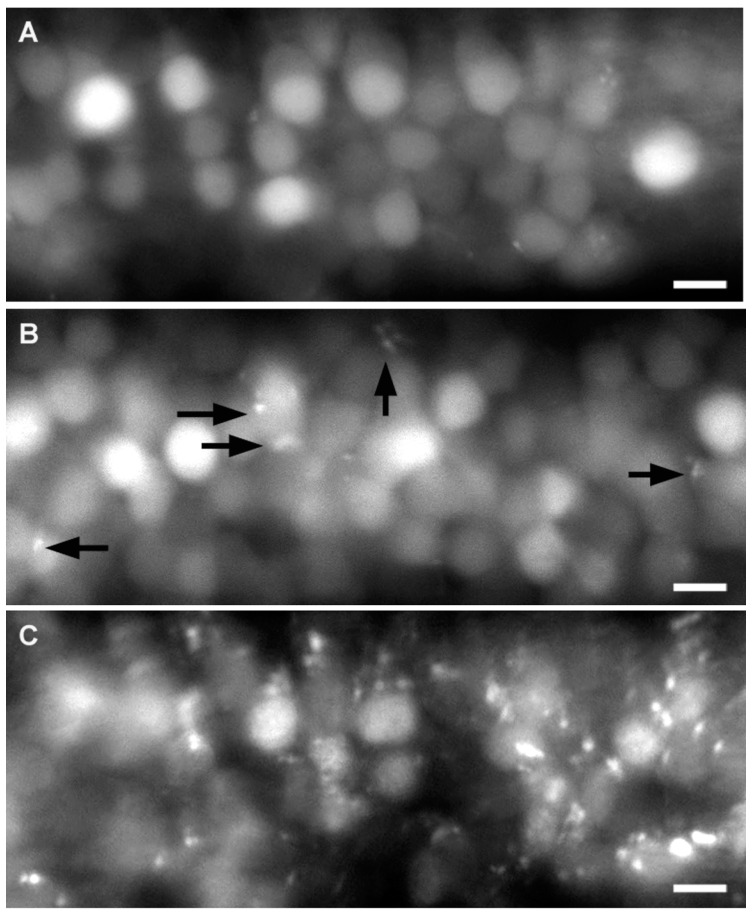
Effect of cyprodinyl and A6 on the central nervous system of *nbt-dsred* larvae. 1-dpf embryos were exposed to the solvent alone (**A**); to 20 µM cyprodinyl (**B**); or to 20 µM A6 (**C**), and their spinal cord neurons were visualized after two days of incubation. At least nine larvae were examined for each condition, with very similar results. Arrows indicate fluorescent inclusions. Scale bars: 10 μm.

The presence of behavioral defects after exposure to either A6 or cyprodinyl led us to examine possible effects on the central nervous system, using the *nbt-dsred* line where all neurons express a red fluorescent marker. We first checked that there is no difference in survival between wild type and *nbt-dsred* fish (data not shown). We observed that spinal cord neurons are homogeneously labeled in untreated larvae (Figure 2A), whereas larvae exposed to 20 µM A6 for two days, from 1 to 3 dpf, show obvious signs of degeneration (Figure 2C). Larvae exposed to 20 µM cyprodinyl exhibit a few fluorescent inclusions (Figure 2B, arrows), suggesting that both compounds might have a toxic effect on neurons, although the effect of A6 is much more dramatic than that of cyprodinyl, consistent with the observation that survival at 20 µM is 60% for cyprodinyl, but only 20% for A6. These neural abnormalities might be a result of lethality, however, rather than its cause, and therefore we did not attempt to quantify these defects, but rather decided to quantify the effects of much lower doses of A6 on a particular sensory system, the lateral line system.

### 2.3. Effect of A6 on the Lateral Line System

**Figure 3:**
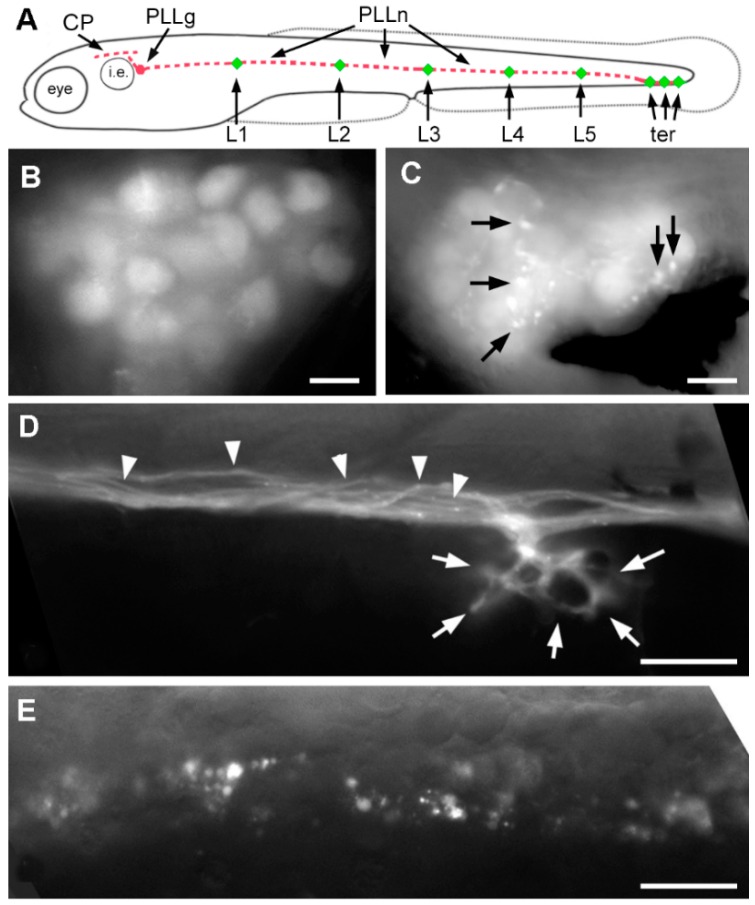
Effect of A6 on the posterior lateral line (PLL) system. (**A**) Scheme of a 4-dpf larva showing the posterior lateral line ganglion (PLLg) just posterior to the otic vesicle (i.e., inner ear), its central projection (CP) extending along the hindbrain, and the peripheral nerve (PLLn) extending along the horizontal myoseptum towards the sensory organs (in red), neuromasts L1–L5 and terminal neuromasts, ter (in green); (**B**) Lateral line ganglion in the *nbt-dsred* line; (**C**) As in (**B**), in a larva that had been exposed to 20 µM A6 from 1 to 3 dpf; (**D**) Lateral line nerve and branch to a neuromast in the *nbt-dsred* line; (**E**) Lateral line nerve in a larva that had been exposed to 20 µM A6 from 1 to 3 dpf. Arrows in (**C**) indicate fluorescent inclusions. Arrowheads in (**D**) point to individual axons within the PLL nerve; arrowheads show axonal terminations surrounding the mechanosensory hair cells of a neuromast; Scale bars: 10 µm.

In order to better define neurotoxicity in zebrafish larvae, we concentrated on the mechanosensory lateral line system. This system comprises a set of superficial sense organs, the neuromasts, which are innervated by sensory neurons present in cranial ganglia. We focused on the posterior lateral line (PLL, Figure 3A) which comprises all neuromasts present on the trunk, tail and caudal fin. PLL neurons are clustered in a ganglion located just posterior to the inner ear, and extend a peripheral axon towards their target neuromasts, and a central axon into the hindbrain. The peripheral axons form a nerve that runs along the horizontal myoseptum, right under the epidermis, thus greatly facilitating the observation of abnormalities [22]. Although the PLL comprises about 50 neuromasts in juvenile zebrafish, and several hundred in old adults [23], the system is much simpler in 6 dpf larvae as it comprises 5–6 neuromasts regularly spaced along the body (L1–L5) and 2–3 terminal neuromasts (Figure 3A).

We observed that exposure to 20 µM A6 for 2 days, from 1 to 3 dpf, results in numerous inclusions in the cell bodies of the PLL sensory neurons (Figure 3C), although such inclusions are seldom observed in untreated larvae (Figure 3B). At the level of the PLL nerve, individual axons are readily detected in untreated larvae (Figure 3D, arrowheads), as well as axonal branches surrounding individual mechanosensory hair cells (Figure 3D, arrows). In A6 treated larvae, however, the nerve appears to be massively degenerating, and the branches innervating hair cells are absent altogether (Figure 3E). Given its simplicity and accessibility, we decided to use the lateral line system to look for neurotoxicity of A6 at very low doses.

### 2.4. Neural Accumulation of A6 in the Lateral Line System

**Figure 4:**
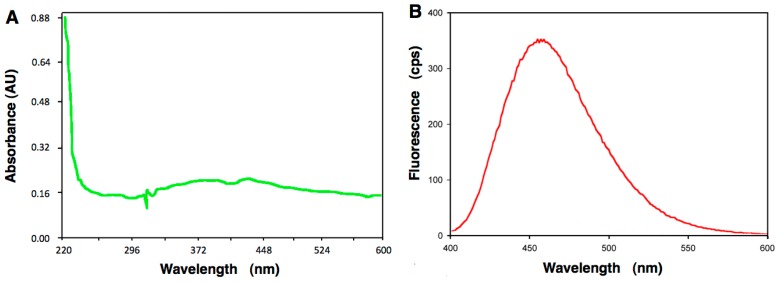
Fluorescent properties of A6. (**A**) Absorption spectrum; (**B**) Fluorescence spectrum after excitation at λ = 372 nm.

α-Terthienyl, the vegetal molecule from which A6 is derived, was originally isolated based on its unusual blue-fluorescent properties [14]. This led us to examine whether A6 would also be fluorescent. Spectral analysis revealed a broad peak of absorption from 320 to 500 nm (Figure 4A). After excitation at λ = 372 nm, we observed a strong fluorescent signal with a maximal emission at 460 nm (Figure 4B). This fluorescent signal is nearly identical to the one of α-terthienyl (λ_exc_ = 361 nm and λ_em_ = 428 nm, data not shown).

**Figure 5:**
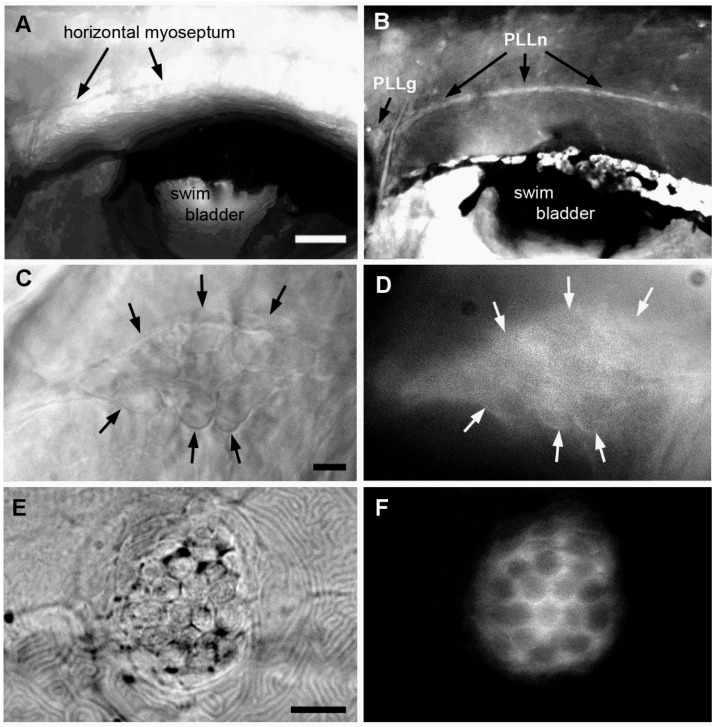
Accumulation of A6 in the lateral line system of embryos that had been incubated in 50 nM A6 from 1 to 6 dpf. (**A**,**B**) Anterior trunk region; (**C**,**D**) Lateral line ganglion; (**E**,**F**) Neuromast L2; (**A**,**C**,**E**) Bright field; (**B**,**D**,**F**) Fluorescence. Arrows in (**C**,**D**) outline the PLL ganglion. Scale bars: (**A**) 100 μm, (**C**,**E**) 10 μm.

We then relied on fluorescence microscopy to determine whether exposure to A6 leads to accumulation of the compound in the larval body, and if so, to determine its distribution. We found that A6 is readily detected in the PLL ganglion and nerve (Figure 5B,D), but not in surrounding tissue. We also found that A6 is taken up by the mechanosensory hair cells of the neuromasts (Figure 5F). Hair cells share many features with neurons, including electric excitability and presence of presynaptic vesicles. The fact that both neurons and hair cells take up A6 preferentially suggests, therefore, that neurons may be primary targets of A6.

### 2.5. Effect of Low Doses of A6 on Axon and Hair Cell Regeneration

**Figure 6:**
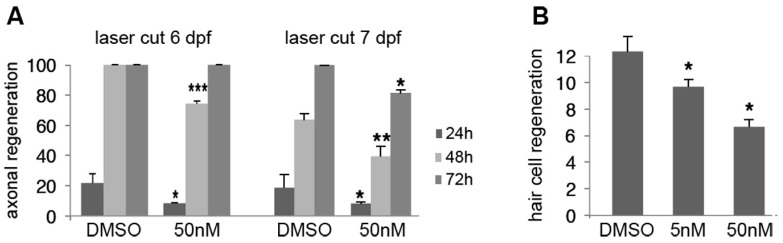
Effect of A6 on axonal and hair cell regeneration. (**A**) Extent of axonal regeneration expressed as % of neuromasts that had been reinnervated after the nerve was cut just posterior to L1 at 6 dpf or 7 dpf. Larvae were exposed to A6 between 1 and 5 dpf (**left panel**) and between 1 and 6 dpf (**right panel**), and reinnervation was examined 1, 2 and 3 days after the cut; (**B**) Extent of hair cell regeneration expressed as number of hair cells present 1 day after complete ablation of all hair cells through copper sulfate treatment at 7 dpf. Larvae had been exposed to A6 between 1 and 6 dpf. Three independent experiments were performed for laser cut, *n* = 18 larvae for each condition, and three independent experiments were performed for regeneration experiment. Results are given as mean values ± standard error of mean (SEM). Data analysis: see Materials and Methods, Section 4.10. Symbols: * *p* < 0.05, ** *p* < 0.01; *** *p* < 0.001.

We examined whether sub-micromolar concentrations of A6 affect not only neuron survival, but also axonogenesis by examining the regenerative capabilities of PLL axons after a nerve cut [24]. The PLL nerve was visualized in *nbt-dsred* larvae that had been continuously exposed to various concentrations of A6 from 1 dpf on, and cut with a laser beam just posterior to L1 at either 6 or 7 dpf. This leads to complete degeneration of the nerve distal to the cut after a few hours. The neuromasts are not affected by this treatment, and can be visualized by labeling with the hair cell-specific dye, DiAsp [24] (see Materials and Methods, Section 4.7). The level of regeneration (number of reinnervated neuromasts vs. total number of neuromasts posterior to L1) was subsequently measured one, two and three days after the cut. Figure 6A reveals that axonal regeneration is significantly impaired when the larvae were bathed in 50 nM A6, half of the concentration where survival after 4 days of exposure is 80% (Figure 1D).

Because A6 accumulates preferentially in the mechanosensory hair cells of the neuromasts (Figure 5F), we also examined whether this compound might affect hair cell regeneration. Hair cells in neuromasts are readily killed by various treatments such as aminoglycosides antibiotics, e.g., neomycin [25], or copper sulfate [26]. Contrary to the hair cells in the mammalian cochlea, however, hair cells in neuromasts are quickly regenerated by other cell types present in the neuromasts [27]. In fact, neuromast hair cells are continuously replaced by new hair cells over the normal life of the fish [28].

Hair cells were killed by copper sulfate treatment at 7 dpf in larvae that had been continuously exposed to various concentrations of A6 from 1 dpf on. The number of regenerated hair cells was counted on the next day. There is a significant impairment of hair cell regeneration at 50 nM A6, and even at the extremely low concentration of 5 nM (Figure 6B).

### 2.6. A6 Down-Regulates Gene Expression

**Figure 7:**
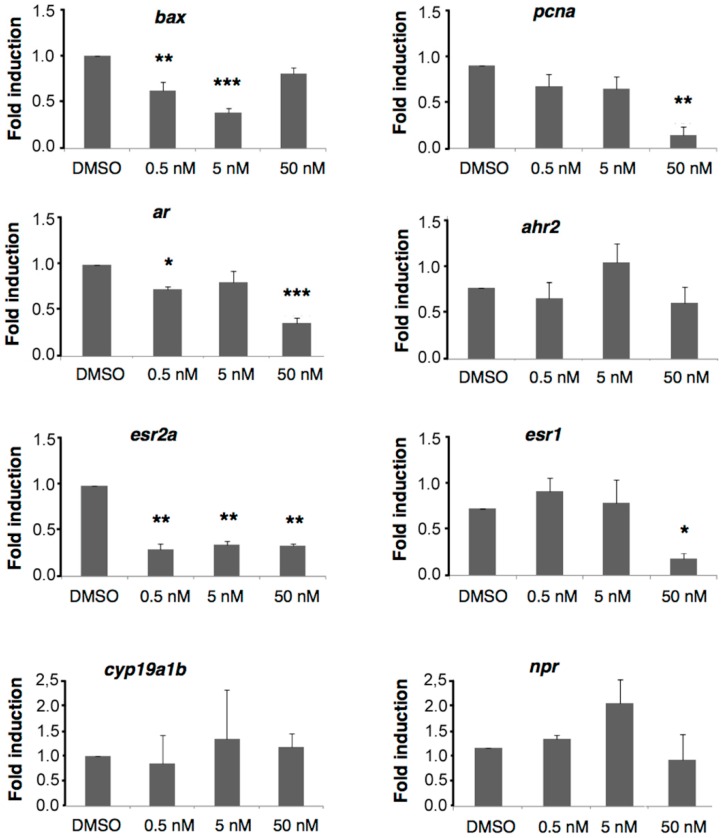
Relative gene expression of *bax*, *pcna*, *ar*, *ahr2*, *esr2a*, *esr1*, *cyp19a1b* and *pr* after exposure to A6. Relative transcript abundance was quantified by real-time reverse transcription PCR; the results are expressed as fold changes (log2) in mRNA abundance as compared to control values, as determined using the 2^ΔΔ*C*t^ method. Results are given as mean values ± SEM (*n* = 6–9 replicates). Asterisks indicate significantly lower expression than control (* *p* < 0.05, ** *p* < 0.01, *** *p* < 0.001).

In order to obtain insights on the molecular mechanisms triggered by A6, we examined whether this compound has an effect on the transcription of eight candidate genes, including *bax* (BCL-2 associated X) and *pcna* (proliferating cell nuclear antigen) involved in cell survival and proliferation, respectively. The transcripts of *bax*, a gene known for its pro-apoptotic role in cells [29] are significantly down-regulated at 0.5 and 5 nM but not at 50 nM, suggesting that low doses of A6 might favor cell survival (Figure 7). On the other hand, the transcripts of the cell proliferation marker *pcna* are massively down-regulated at 50 nM, suggesting that a decrease in cell proliferation could be the major factor in the reduction of hair cell regeneration observed at this concentration. The slight decrease in hair cell regeneration observed at 5 nM A6 could be related to the slight, though not statistically significant, decrease in *pcna* expression at this concentration, or to yet other effects of A6 at low doses.

Anilinopyrimidines were previously reported to act on androgen receptors and aryl hydrocarbon receptors [20]. We analyzed the expression of the corresponding genes, *ar* and *ahr2*, and found that only the transcripts of *ar* are down-regulated at 50 nM A6 (Figure 7), with a barely significant effect at 0.5 nM.

Many studies have shown that estrogen receptors (ER) are targeted by pesticides that act as endocrine disruptors, and are also implicated in proliferation processes. As ERα is regulated by its natural ligand [30], we examined whether A6 might affect the expression of either or both ER receptors. We measured the expression of *esr1* (coding for ERα) and of *esr2a* (coding for ERβ) and found that A6 down-regulates *esr2a* at all concentrations tested down to 0.5 nM, whereas it affects the transcription of *esr1* at 50 nM but not at lower concentrations (Figure 7).

We also looked at the expression of *cyp19a1b*, the gene coding for aromatase B, a protein known to be up-regulated by estrogens [31] and of *pr*, the gene coding for the progesterone receptor [32]. Both transcripts remained unaffected by A6 at all concentrations tested, suggesting that the effect of A6 at low doses may act, at least partly, through non-canonical endocrine signaling pathways.

### 2.7. Molecular Modeling Studies of A6 and Cyprodinyl Binding on Estrogen Receptors

Since our data show that A6 targets the expression of genes coding for ER, which is also regulated by their natural ligand, we assessed a possible interaction between A6 and ER through molecular modelling studies [33]. We performed docking studies of A6, of cyprodinyl and of 17-α-ethinylestradiol (EE2), an analog of the natural estrogenic ligand, on the ligand binding domain (LBD) of the crystal structure of human ERα (Protein Data Bank ID, 4MGA) [33] using Genetic Optimization for Ligand Docking (GOLD) software (version 5.2, The Cambridge Crystallographic Data Centre, Cambridge, UK). The compounds were ranked according to their respective scores.

**Figure 8:**
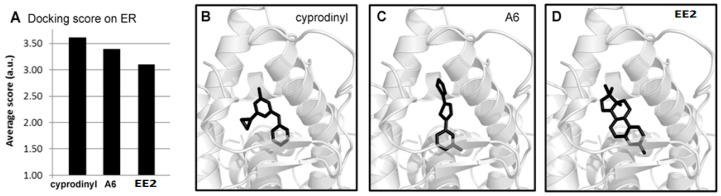
Putative binding of A6 and cyprodinyl to the ligand binding site of estrogen receptors (ER) predicted by the Genetic Optimization for Ligand Docking (GOLD) docking software. (**A**) Estimated binding efficiencies of cyprodinyl, A6, and the natural ligand analog 17-α-ethinylestradiol (EE2) for the ligand binding site of the ER (Protein Data Bank ID, 4MGA); (**B**–**D**) Graphical representation of cyprodinyl, A6, and EE2 docked on the ER ligand binding domain (Protein Data bank ID, 4MGA). Ligands are shown in black, the ligand binding domain in grey. All models were rendered in PyMOL.

The results (Figure 8A) reveal that A6 is predicted to bind to ER, with a score higher than that of EE2 (3.4 vs. 3.0). Moreover, A6, cyprodinyl and EE2 were found to dock at similar positions of the LBD of human ER, suggesting a competitive binding of these molecules (Figure 8B–D). Taking into account the estimated binding affinity (computed scores) and the experimental evidence that A6 modulates gene expression, one would expect the natural ligand to be displaced by A6 in the ER.

## 3. Discussion

Focusing on the impact of very low doses of pollutants in aquatic systems is not the traditional way of evaluating the ecotoxicity of a given pollutant, as standardized tests determine only the effects of a compound at lethal or near-lethal concentrations. Assessing the effect of micropollutants is, however, essential to understand the long-term biological effects of widely used pesticides. In this context, the effect of low doses of naturally occurring pesticides, and of their derivatives, is an important aspect in assessing their superiority over industrial compounds [34].

In this study we evaluate the toxicity to vertebrates of a potentially useful pesticide, A6, which was derived from the naturally occurring nematicide/insecticide α-terthienyl, through substitution of one of the three thienyl rings with a pyrimidine. We show that exposure of zebrafish larvae to A6 induces larval mortality at the relatively low concentration of 0.5 μM, with marked effects on the nervous system, both central and sensory. Similar lethalities have been reported for other pesticides tested on zebrafish larvae [35,36,37]. Using the well-defined and easily accessible lateral line sensory system as an assay, we observed that A6 affects axonal regeneration at the low dose of 50 nM, suggesting that this compound may have serious long-term effects on neural plasticity and repair at these low doses.

We further show that A6 has significant adverse effects on hair cell regeneration at the very low concentration of 5 nM. This result may not be significant for human welfare, as the loss of auditory hair cells (and subsequent auditory damage) is irreversible in mammals, and therefore the mammalian auditory system cannot be repaired through hair cell regeneration anyway. Nevertheless, our observations that A6 preferentially accumulates in lateral line hair cells and is neurally toxic, suggest that the effect of A6 on mammalian auditory hair cells deserves to be examined more closely. Furthermore, the adverse effect documented in the present study may be viewed in the broader context of impaired regeneration of cells and cell components, and there is much to be discussed in this context on the potential impact of chronic exposure to very low levels of toxic compounds.

α-Terthienyl was originally isolated from the petals of the common marigold (*Tagetes erecta*), based on its unusual spectral properties [14]. This compound was later found to be produced in the roots of this plant, and to display an unusual, photosensitive pesticidal activity. Under near-UV light and in the presence of molecular oxygen, α-terthienyl produces singlet oxygen which oxidizes key substrate molecules in living organisms, making it a potent nematicide and insecticide [38]. It may be, therefore, that photo-activated toxicity contributes to the neurotoxic effect of A6. In our experimental set-up, however, larvae were kept in the dark. We believe, therefore, that the major component of A6 neurotoxicity in zebrafish at all doses is not related to the production of singlet oxygen through its bithienyl moiety, but lies in its capability to bind to estrogen receptors.

Based on the presence of a pyrimidine residue in another class of pesticides, anilinopyrimidines which are suspected to be endocrine disruptors [20], we examined whether A6 might have an effect on the expression of genes involved in endocrine signaling. Our results show that A6 down-regulates the expression of the genes coding for the two estrogen receptors, ERα, ERβ, and for AR, the androgen receptor. This down-regulation is observed at concentrations as low as 0.5 nM for ERβ and AR.

The activation of ER receptors by their natural ligand, estradiol, induces the transcription of target genes such as *cyp19a1b*, coding for aromatase B, an enzyme involved in the transformation of androgens into estrogens [31]. We observed, however, that the down-regulation of both estrogen receptors by A6 has no impact on the expression of *cyp19a1b*. This could be due to a number of reasons; for example, it may be that the level of expression of *cyp19a1b* is regulated by other estrogenic pathways. Whatever the reason, the fact that a concentration of 50 nM A6 results in a significant decrease in axonal and hair cell regeneration, but has no effect on aromatase, suggests that at least part of the defects due to exposure to doses of A6 as low as 5 nM do not result from this particular estrogenic pathway.

An alternative, or additional, target of A6 might be the G-protein-coupled estrogen receptor (Gper, formerly known as GPR30), a relatively recent addition to the large family of G protein-coupled receptors [39]. The binding of estrogen to this receptor activates cAMP signalling cascades that enhance proliferation, growth, and cell survival [40,41]. The *gper* gene is highly expressed in rat neurons [39], and the receptor was shown to be localized at the plasma membrane in primary cultures of rat hippocampal neurons. In hypothalamic neurons, however, Gper is distributed throughout the cytoplasm and in the perinuclear region [42]. It was shown recently that in zebrafish, *gper* is expressed in various regions of the developing brain, and that *gper* knockdown results in growth retardation, and in morphological defects in the developing brain, with decreased proliferation of brain cells and abnormalities in the development of sensory and motor neurons [43]. These findings are entirely consistent with our results on decreased regeneration of hair cells and sensory axons after treatment with A6, although they do not yet provide a mechanistic basis for these defects. Whether A6 directly binds Gper, and with what effects, should definitely be studied in the future. For the moment, however, the crystal structure of this receptor is not known, and docking analyses can therefore not be performed yet.

Our results raise the possibility that some pesticides may simultaneously affect endocrine disruption, and be neurotoxic, by acting on different receptors or through different pathways [44]. A similar conclusion was drawn from studies of PBDE-47, a polybrominated diphenyl ester used as flame retardant in polyurethane foams, which was shown to be genotoxic, neurotoxic, and an endocrine disruptor [45]. In this context, it may be interesting to determine to what extent other compounds known as endocrine receptors also show some neurotoxicity, and conversely, whether compounds characterized as neurotoxic may turn out to affect endocrine signaling as well, at least to some extent. If this were the case, then one should consider whether the less visible phenotype is necessarily less important for human health.

All pesticides are susceptible to have adverse effects on living organisms, and biopesticides do not escape this rule. Understanding their mechanism of action in order to anticipate their potential impact on human health is, therefore, crucial. This understanding must take into account the fact that pesticides can act through various mechanisms, and that focusing exclusively on their most visible effects may be misleading about their long-term effects: as nicely expressed in Kenneth Koch’s poem, “One train may hide another”.

## 4. Materials and Methods

### 4.1. Ethics Statement

Experiments were performed in the zebrafish facility platform based in our laboratory Inserm U1198 following the National and European ethic guidelines for animal well-being and the European Convention for the Protection of Animals used for Experimental and Scientific Purposes. The present study was approved by INSERM and Montpellier University (agreement #A34-172-37, 10 April 2013).

### 4.2. Fish Strains and Treatment of Embryos

Zebrafish (*Danio rerio*) were handled according to standard procedures [46]. Adult fish were maintained at 28 °C, with a circadian rhythm of 14 h of light and 10 h of darkness, in a recirculation, flow-through system (Aquatic Habitats, Apopka, FL, USA) where tank water is constantly filtered and UV-sterilized. Fish were fed twice daily. Embryos were obtained from pair matings. One male and one female were placed in the afternoon in the two compartments of 500 mL containers with plastic mesh bottoms that allow the fertilized eggs to fall in an underlying container. The partition between male and female was removed next morning at the onset of the light period, usually resulting in spawning over the next 30 min.

Embryos were incubated in tank water at 28 °C. The AB line was used as wild-type. Neurons were visualized in the *nbt-dsred* line [47], and hair cell regeneration was assessed in *Brn3c::GFP* larvae, where all hair cells express the green fluorescent protein GFP [48]. Embryos were dechorionated at 1 dpf prior to all experimental exposures, and subsequently maintained in the dark in 6-well plates, with 5–10 embryos in 2 mL of solution for each well. As egg lays were sometimes of lesser quality, depending on the age of the parents or other factors, we processed only batches of eggs where all embryos had developed normally at 1 dpf. Under this condition we observed no lethality in the control groups up to 5 dpf.

### 4.3. Pesticides

Compound A6, also known as pyrimidino bithiophene [15], with the chemical formula 2-(2′-thienyl)-5-(4′′-(2′′-aminopyrimidyl)thiophene) was purchased from Maybridge (Cornwall, UK). Cyprodinyl (4-cyclopropyl-6-methyl-*N*-phenyl-pyrimidinamine), mepanipyrim (4-methyl-*N*-phenyl-6-(1-propynyl)-2-pyrimidinamine) and pyrimethanil (2-anilino-4,6-dimethylpyrimidine) were purchased from Sigma (Saint-Quentin, France). Stock solutions of all pesticides were prepared at a concentration of 10 mM in pure DMSO. A6 was heated at 80 °C for 10–20 min to reach complete disolution. Intermediate stock solutions were prepared in distilled water in order not to exceed a concentration of DMSO of 0.1% in survival experiments, and 0.01% in axonal and hair cells regeneration experiments.

### 4.4. Survival Curves and Behavioral Observations

Dechorionated embryos were maintained in 6 well-plates in different pesticides (A6 or anilinopyrimidines) at final concentrations varying between 0.1 to 50 µM during 4 days (1–5 dpf). Control groups were treated with DMSO (0.1%). For behavioral observations, embryos were exposed to pesticides as described above, put individually in the wells of a 6-well plate and into a ZebraBox recording system (Viewpoint, Lyon, France) and allowed to settle for 10 min. They were then continuously monitored for the next 10 min using using ViewPoint Application manager and ZebraLab tracking softwares for locomotor activity studies [49].

### 4.5. Absorption Spectroscopy of A6 and Fluorescence Studies

A stock solution of A6 at 1 mg/mL (3.9 mM) was diluted in 50 mM MES (2-morpholinoethanesulfonic acid) buffer pH 6.0 at a final concentration of 4 mg/mL (15.5 mM), and the absorption spectrum was recorded from 220–600 nm using a spectrophotometer Specord 250 (Analytikjena, Jena, Germany). The fluorescence spectrum was recorded following excitation at 372 nm, using a fluorimeter FluoroMax2 (JobinYvon Spex, Tokyo, Japan) between 400–600 nm, using a bandwidth of 16 nm. More than 20 recordings were used for establishing the fluorescence spectrum.

### 4.6. Neural Accumulation

For live imaging, embryos were anesthetized using tricaine (3-amino benzoic acid ethylester, Sigma, 4 mg/mL in 20 mM Tris at pH 7, diluted 25× in tank water just before use) and mounted on depression slides in 0.5% agar in tank water. Anesthetized embryos were observed under a Zeiss AxioImager microscope using the Zeiss filter set 02 for UV fluorescence. No fluorescence can be detected in untreated AB larvae under these conditions. Image stacks were processed with Fiji. The number of fish embryos observed for a given concentration was superior to 15 from three independent experiments.

### 4.7. Axonal Regeneration

The PLL nerve of *nbt-dsred* larvae exposed to A6 was cut with a Micropoint laser system (Photonics Instruments, Pittsfield, MA, USA) adapted to a Zeiss Axioplan 2 microscope equipped with a 40× water immersion objective, using coumarin 440 nm, 5 mM in methanol, as a laser medium. For visualizing axonal regeneration, the larvae were first incubated for 5 min in 4-(4-diethylaminostyryl)-*N*-methylpyridinium iodide (DiAsp 2 μg/mL, Sigma), a vital dye taken up by the hair cells of neuromasts [24]. Larvae were then anesthetized and mounted as described above, and the number of re-innervated neuromasts (as detected by the presence of innervating axonal branches) was assessed with a Zeiss Axioimager equipped with a 63× water immersion objective, and a Coolsnap camera. The % regeneration refers to the number of re-innervated, relative to the total number of neuromasts distal to the cut. Three independent experiments were performed for laser cut, *n* = 18 larvae for each condition, and three independent experiments were performed for regeneration experiment.

### 4.8. Hair Cell Regeneration

Embryos of the *Brn3c::GFP* line were exposed to the experimental solutions between 1 and 7 dpf, and then incubated in 10 mM CuSO_4_ for 2 h to kill all neuromast hair cells. Hair cell regeneration was observed on the following day with a Zeiss Axioimager equipped with a 63× water immersion objective, and a Coolsnap camera. Three independent experiments were performed, *n* = 30 larvae for each condition.

### 4.9. Determination of Gene Expression Levels by Quantitative Real-Time PCR

#### 4.9.1. Embryo Exposure

Fertilized wild type eggs were exposed from 1 to 6 dpf to A6 (0.5, 5 and 50 nM) or to solvent DMSO control (0.01% *v*/*v*). Each experimental group consisted of 80 embryos treated in 100 mL of the solution. Embryos were kept in an incubator at 28 °C.

#### 4.9.2. RNA Isolation

Total RNA was extracted from pools of 60 larval heads using RNeasy Mini Kit (Qiagen, Courtaboeuf, France) following the manufacturer’s protocol. RNA concentrations and quality were analyzed using a NanoDrop 8000 spectrophotometer (Thermo Scientific, Wilmington, DE, USA).

#### 4.9.3. qRT-PCR Analyses: *esr1*, *esr2a*, *ar*, *npr*, *ahr2*, *cyp19a1b*, *pcna*, *bax* and *ef1a*

mRNAs were reverse-transcribed and amplified by real-time PCR using a Light Cycler detection system (Roche, Basel, Switzerland). Amplification was undertaken using a Platinum SYBR Green qPCR SuperMix-UDG kit (Fischer Scientific, Illkirch, France) according to the instructions provided by the manufacturer. The qPCR experiments were performed in collaboration with the qPCR platform of the University of Montpellier following the MIQE (Minimum Information for Publication of Quantitative real-Time PCR Experiments) guidelines [50]. The Δ*C*_t_ value was derived by subtracting the threshold cycle (*C*_t_) value of the housekeeping gene (elongation factor 1α, *ef1a* also named *elfa*), which served as an internal control, from the Ct value of the target gene, respectively. For each target genes, the Δ*C*_t_ values were calibrated against the control Δ*C*_t_ values (ΔΔ*C*_t_) and, the relative linear amount of target molecules relative to the calibrator was calculated as 2^ΔΔ*C*t^. The reference gene was chosen based on published data showing that among eight common housekeeping genes tested, the expression of *ef1*a is one of the most stable during the development of zebrafish and across tissue types [51], as well as following chemical treatment with endocrine disruptors and solvent [52], *ef1*a was therefore chosen in our qPCR experiments as the reference gene. Each experiment was performed twice independently at least in triplicate. Oligonucleotide primers used for cDNA amplification were as Table 1.

### 4.10. Data Analysis

Survival curves were done using Prism software (version 5.0a, GraphPad Software, La Jolla, CA, USA). For each pesticide, the difference between curves established at various concentration was tested using the non-parametric Mantel-Cox test, with a probability of 0.05 defined as a significant difference (* *p* < 0.05).

The Student’s *t*-test was used for statistical data analysis of the PLL nerve regeneration, with a probability of 0.05 defined as a significant difference (* *p* < 0.05, ** *p* < 0.01, *** *p* < 0.001).

ANOVA one-way test for the analysis of the variance followed by Tukey’s multiple comparison of means HSD (honest significant difference) post-hoc test (Tukey’s HSD) was performed to analyze the data of hair cells regeneration experiments. Differences were considered statistically significant when *p* < 0.05 (* *p* < 0.05, ** *p* < 0.01, *** *p* < 0.001).

For each gene tested, the qPCR results are given as means ± standard error of mean (SEM). The statistical analyses given for qPCR were performed using ANOVA one-way test followed by Tukey’s HSD. Q-PCR analyses were conducted using R software. Differences were considered significant at *p* ≤ 0.05 (* *p* < 0.05, ** *p* <0.01, *** *p* < 0.001). For each gene tested, a normalized histogram was established by dividing each values obtained at 0.5, 5 and 50 nM of A6, to the control value (DMSO).

### 4.11. Molecular Modeling Studies

Binding site prediction of the three compounds was achieved using GOLD program [53] by applying 50 runs of genetic algorithm. The binding search was centered on His291 (CE atom) belonging to the ligand binding domain (LBD) of ER (PDB, 4MGA), with a radius of search of 15 Å. The different docking poses were analyzed by the clustering method (complete linkage) from the rmsd (Root-Mean-Square Deviation) matrix of ranking solutions. Docking poses were classified according to their respective score calculated by the goldscore scoring function. To compare the compounds differing in molecular weight, scores were normalized according to their number of heavy atoms (NHA). A second docking program, Plants [54], confirmed the GOLD results. The visualization of docking poses of the compounds in the crystal structure of human ER was carried out using the PyMOL Molecular Graphic System (version 1.3, Schrödinger, LLC, Cambridge, UK).

## 5. Conclusions

We show that A6, a pesticide derived from the naturally-occurring α-terthienyl and structurally related to anilinopyrimidines, is a potent neurotoxin for zebrafish larvae. Nanomolar doses of A6 have a clear effect on axonal and mechanosensory hair cell regeneration. We found that A6 affects the expression of genes reflecting estrogen receptor activation, consistent with previous reports that anilinopyrimidines act as endocrine disruptors. Canonical targets of endocrine signaling are not affected, however, suggesting that the neurotoxic effect of A6 may be due to the binding of this compound to a recently identified, neuron-specific estrogen receptor. This result suggests that binding of pesticides to estrogen receptors may have direct neurotoxic effects, raising the question of the relationship between endocrine disruption and neurotoxicity, and suggesting that pesticides can act through various mechanisms We conclude that focusing exclusively on the most visible effects of pesticides at lethal doses may be misleading about their long-term effects at very low doses.

## Figures and Tables

**Table 1:** Primers used for qRT-PCR analyses.

Gene Name	Genebank Number	Reverse (rev) and Forward (fw) Primers
*ahr2*	Genbank accession number 105762.2	(fw) 5′-GAAGAAGCCCGTTCAGAAAA-3′ (rev) 5′-GGGTTGGATTTCACACCATC-3′
*ar*	Genbank accession number 1083123.1	(fw) 5′-CACTACGGAGCCCTCACTTGCGGA-3′ (rev) 5′-GCCCTGAACTGCTCCGACCTC-3′
*bax*	Genbank accession number BC55592	(fw) 5′-GAGCTGCACTTCTCAACAACTTT-3′ (rev) 5′-CTGGTTGAAATAGCCTTGATGAC-3′
*cyp19a1b*	Genbank accession number 131642.1	(fw) 5′-TCGGCACGGCGTGCAACTAC-3′ (rev) 5′-CATACCTATGCATTGCAGACC-3′
*ef1a*	Genbank accession number 131263.1	(fw) 5′-AGCAGCAGCTGAGGAGTGAT-3′ (rev) 5′-CCGCATTTGTAGATCAGATGG-3′
*esr1*	Genbank accession number 152959.1	(fw) 5′-CTGGAGATGCTGGACGCTCA-3′ (rev) 5′-GCTGCAGCTCCTCCTCCTGG-3′
*esr2a*	Genbank accession number 180966.2	(fw) 5′-GATCCTGCTCAACTCTAATAAC-3′ (rev) 5′-CCAGCAGATTCAGCACCTTCCC-3′
*pcna*	Genbank accession number BC49535.1	(fw) 5′-CTCACAGACCAGCAACGTCG-3′ (rev) 5′-GGACAGAGGAGTGGCTTTGG-3′
*pr*	Genbank accession number EF155644.1	(fw) 5′-GAGCAATGATCAGCTGAGAAGG-3’ (rev) 5′-TCCAGAGGAACAGTGTTGAGG-3′

## Supplementary Materials

Supplementary materials can be found at www.mdpi.com/1422-0067/17/12/2137/s1.
