# Supplementary material for: Neurotoxicity of a Biopesticide Analog on Zebrafish Larvae at Nanomolar Concentrations
Source: Int J Mol Sci. 2016 Dec 19;17(12):2137. doi: 10.3390/ijms17122137 (PMC5187937; doi:10.3390/ijms17122137)
Supplement: Supplementary file 1 [file ijms-17-02137-s001.pdf]

Int. J. Mol. Sci. **2016**, *17*, 2137; doi:10.3390/ijms17122137

Supplementary Materials: Neurotoxicity of a Biopesticide Analog on Zebrafish Larvae at Nanomolar Concentrations

Ahmed Nasri, Audrey J. Valverde, Daniel B. Roche, Catherine Desrumaux, Philippe Clair, Hamouda Beyrem, Laurent Chaloin, Alain Ghysen and Véronique Perrier

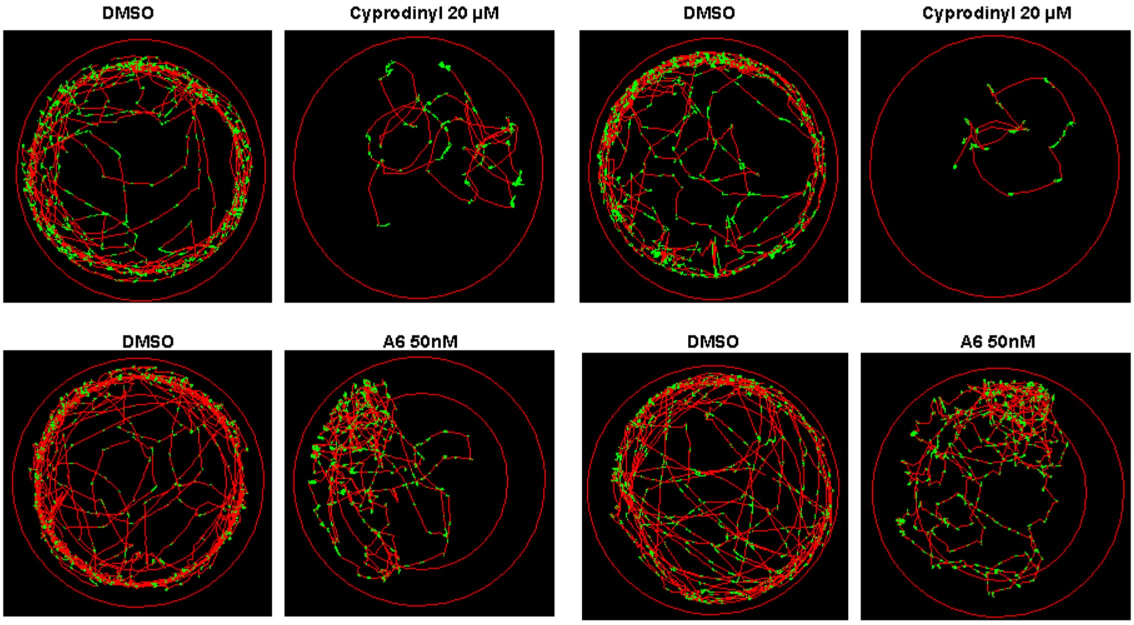

Figure S1. Larval behavior. Larvae are put individually in an observation chamber and their movements are continuously recorded over the next 10 min. Red lines reflect stretches of swimming longer than 2 mm, green lines reflect small movements (0.5–2 mm), below 0.5 mm the larva is considered inactive. The figure shows four representative tracks from 15 larvae exposed to either cyprodinyl 20 µM (**upper row**) or A6 50 nM (**lower row**).
